# SARS-CoV-2 (COVID-19) pneumonia patient treated with two doses of infliximab within 2 weeks for acute severe ulcerative colitis: A case report

**DOI:** 10.1097/MD.0000000000028722

**Published:** 2022-01-28

**Authors:** Marouf Alhalabi, Kamal Alaa Eddin, Fadwa Ali, Ahmad Abbas

**Affiliations:** Gastroenterology Department of Damascus Hospital, Damascus, Syria.

**Keywords:** necrosis factor antagonists, case report, an infectious disease caused by the SARS-CoV-2 virus, infliximab, SARS-CoV-2, ulcerative colitis

## Abstract

**Rationale:**

The ongoing coronavirus pandemic has caused severe acute respiratory syndrome, posing a significant challenge for patients receiving immunotherapy for immune-mediated inflammatory diseases. As of January 2022, immunosuppressants such as tumor necrosis factor inhibitors (anti-TNFα) and azathioprine are inadvisable for an infectious disease caused by the SARS-CoV-2 virus (COVID-19). We continued infliximab as a second induction dose nine days after the onset of COVID-19 symptoms in a patient with acute severe ulcerative colitis.

**Patient concerns:**

We report the case of a 34-year-old male with 6 to 8 times bloody diarrhea, fever, and cramping abdominal pain. Ulcerative colitis was diagnosed 6 months earlier and treated with mesalamine 80 mg/kg/day and azathioprine 2.5 mg/kg/day. The patient had never undergone surgery before. Sigmoidoscopy revealed multiple ulcerations and spontaneous bleeding, and the colon samples tested negative for cytomegalovirus and Clostridium difficile. However, intravenous corticosteroids did not induce remission. A nasopharyngeal swab tested positive for SARS-CoV-2.

**Diagnosis:**

Acute severe ulcerative colitis and SARS-CoV-2 (COVID-19) pneumonia.

**Interventions:**

The second loading dose of infliximab was administered nine days after the diagnosis of COVID-19.

**Outcome:**

The patient completed infliximab induction at a dose of 5 mg/kg at weeks 0, 2, and 6, with no complications.

**Lessons:**

It is unclear whether anti-TNF-α treatment improves or deteriorates COVID-19 patient outcomes, and this case demonstrates that infliximab can be used safely. Current guidelines make a weak recommendation to avoid using anti-TNFα agents in the presence of acute COVID-19 infection. There is an urgent need for research on biologics therapy.

## 1 Introduction

The an infectious disease caused by the SARS-CoV-2 virus (COVID-19) outbreak in December 2019 spread rapidly and caused severe lung damage that can lead to death.^[1,2]^ New cases continue to emerge around the world, during September 2021 Syria experienced a severe wave, with a substantial increase in cases posing a significant threat to public health.^[3]^ According to Ferm et al, COVID-19 infection can cause a variety of symptoms, including gastrointestinal manifestations such as diarrhea (19.8%), nausea (16.6%), loss of appetite (11.8%), vomiting (10.2%), and loss of taste (2.4%).^[4]^ Where all or some of these symptoms are shared with many diseases, including inflammatory bowel diseases, and when these diseases are present, it is difficult to diagnose COVID-19 infection. Higher serum levels of proinflammatory cytokines (TNFα, IL-1, IL-6, and IL-8) in COVID-19 patients cause cytokine storms, which trigger a series of immune responses that damage the corresponding organs.^[5]^ Attempts to treat or relieve infection symptoms rely on suppressing the cytokine storm; dexamethasone, for instance, inhibits severe cytokine storm or the hyper-inflammatory phase in hospitalized COVID-19 patients,^[6–8]^ whereas Neurath argued for an necrosis factor antagonists (anti-TNFα) protective effect in severe COVID-19 patients, but it was not used in COVID-19 treatment, ^[9]^ and both corticosteroids and anti-TNF are essential in the treatment of inflammatory bowel disease.^[10–12]^ We described infliximab-treated acute severe ulcerative colitis (ASUC) with SARS-CoV-2 (COVID-19) pneumonia. Nine days after the onset of symptoms, we administered a second loading dose of infliximab, according to the protocol. The patient completed infliximab induction at 5 mg/kg doses at weeks 0, 2, and 6, with no complications.

## 2 Case presentation

**Figure 1:**
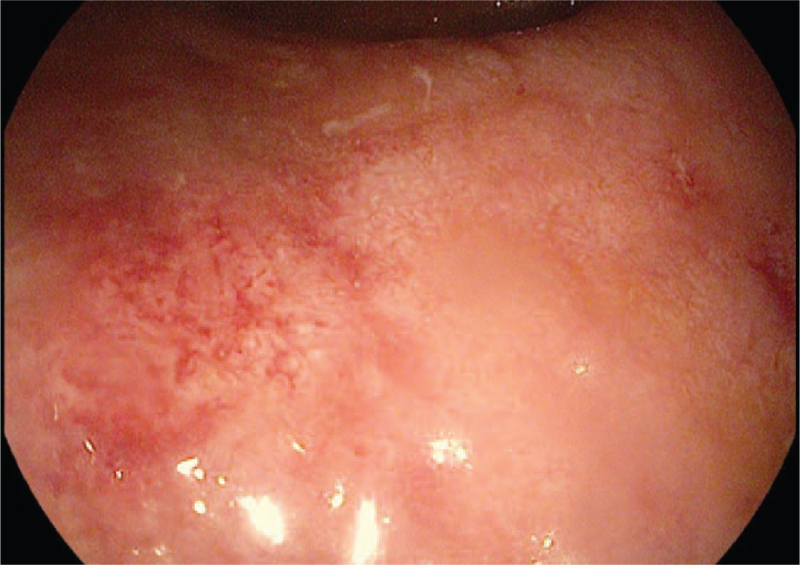
Sporadic ulcers from the sigmoid with areas of spontaneous bleeding.

**Figure 2:**
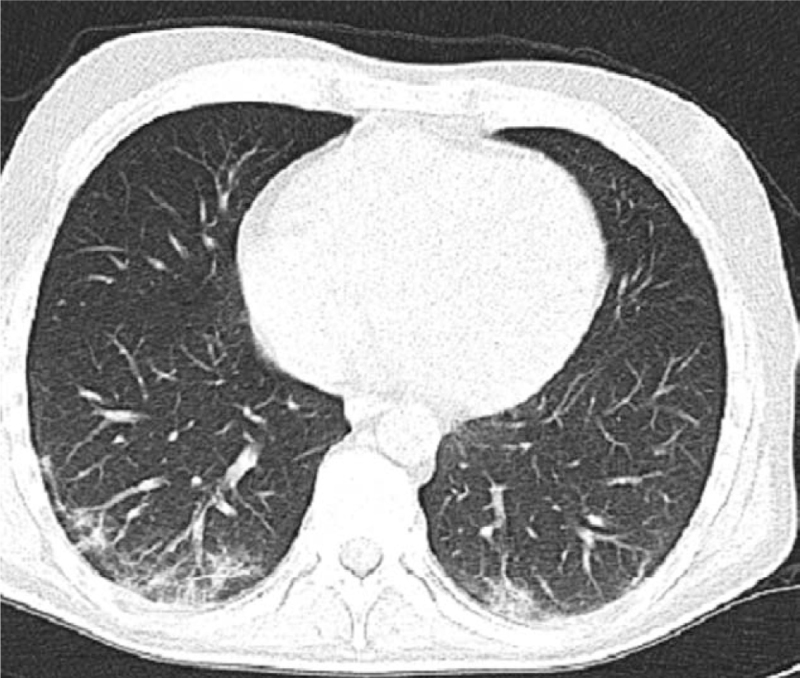
CT shows patchy ground-glass opacities affecting the subpleural lung parenchyma bilaterally, indicating interstitial pneumonia.

We admitted a 34-year-old man with a history of bloody diarrhea 6 to 8 times a day with a fever of 38.9 and cramping abdominal pain. The patient had a history of left-sided colitis, and the diagnosis was established 6 months ago without prior surgery. His past medication included azathioprine 1.5 to 2.5 mg/kg/day,^[10–12]^ and oral mesalamine 2 g/day.^[10–13]^ The patient's adherence to the previous medication was confirmed. On admission, tests revealed leukocytosis, an increase in C-reactive protein (CRP), and an increase in erythrocyte sedimentation rate. COVID-19 rapid antigen test results for IgM and IgG were negative. Stool studies, including ova and parasite microscopy, routine stool cultures,^[14]^ Clostridium difficile toxin testing, and testing for Escherichia coli, Shigella, and Cryptosporidium, all returned negative results. Chest radiography, abdominal radiography, and abdominal ultrasonography findings were normal. Sigmoidoscopy revealed multiple ulcers with spontaneous bleeding extending to the left side of the colon Figure 1, and colon biopsies were negative for cytomegalovirus and Clostridium difficile infections. The patient's Mayo score was 11 points.^[15]^ We started with intravenous fluid and electrolyte replacement, hydrocortisone 100 mg 4 times daily, and low-molecular-weight heparin were administered for thromboprophylaxis.^[10–12]^ The patient did not respond to corticosteroids, and screening for infections as recommended before prescribing biological therapies returned negative results. Infliximab was started at a dose of 5 mg/kg.^[10–12]^ The patient experienced fever, sore throat, dry cough, dyspnea 5 days after starting infliximab, which continued at a frequency of 4 times per day, while bloody diarrhea stopped, and the patient's examination was normal. We performed microbiological studies on a non-invasive respiratory sample,^[16]^ besides, a nasopharyngeal swab was used for reverse transcription-polymerase chain reaction to detect SARS-CoV-2 (COVID-19), which was positive. The patient continued low-molecular-weight heparin (for a hospitalized ASUC), acetaminophen, vitamin D, vitamin C, and zinc intake.^[17,18]^Figure 2 shows a chest computed tomography scan performed three days after the onset of symptoms. The patient had moderate illness, and the symptoms began to improve.^[19]^ On the twelfth day, the patient's fever and shortness of breath disappeared. The patient still complained of diarrhea up to 4 times, without blood, and the partial Mayo score was 4 points. The patient received a second induction dose of infliximab nine days after the onset of COVID-19 symptoms. The patient completed infliximab induction at a dose of 5 mg/kg at weeks 0, 2, and 6, with no complications. Table 1 shows the patients’ test results upon admission and discharge.

**Table 1:** Comparison of test results at admission and on discharge.

Test	At admission	On discharge	Units	Normal value
WBC	10700	8800	mm^3^	4500–10500
CRP	22.3	32	mg/l	0–5
ESR	57	43	mm/hr	Between 0 and 15
Red blood cells	4.40	4.50	mm^3^	(3.7–4.9) × 10^6^
Hemoglobin	13.7	12.30	g/dL	11–14.3
MCHC	34.25	34.17	%	32–36%
MCV	90.91	80	fl	80–94
MCH	31.14	27.33	pg	27–31
Platelets	346 × 10^3^	362 × 10^3^	mm^3^	(150–450) × 10^3^
Sodium	140	139	mmol/l	134–146
Potassium	4.5	4	mmol/l	3.5–5.0
Cl	95	98	mmol/l	95–110
Glucose	72	78	mg/dL	65–110
Urea	7	9	mg/dL	5–45
Creatinine	0.3	0.4	mg/dL	0.3–1.3
INR	1.1	1	1	
ALT/SGPT	10	14	U/L	5–40
AST/SGOT	11	16	U/L	5–40
D-dimer		200	ng/mL fibrinogen-equivalent units (FEU)	≤500

Cl = chloride, CRP = C-reactive protein, D-Dimer = degradation product of crosslinked (by factor XIII) fibrin, ESR = erythrocyte sedimentation rate, INR = international normalised ratio, K = potassium, MCH = mean corpuscular hemoglobin, MCHC = mean corpuscular hemoglobin concentration, MCV = mean corpuscular volume, Na = sodium, SGOT = serum glutamate oxaloacetate transaminase, SGPT = serum glutamate pyruvate transaminase, WBC = white blood test.

## 3 Discussion

Ulcerative colitis is a chronic intestinal disease. It is critical to begin treatment as early as possible, which is known as the treat-to-target management approach.^[20,21]^ Treatment objectives have been established to improve the outcomes. It entails determining an appropriate target, selecting initial therapy, measuring baseline disease characteristics, monitoring treatment progress, and optimizing therapy to achieve the treatment aim.^[21]^ Endoscopic scores such as the Mayo score and the ulcerative colitis endoscopic index of severity, and biomarkers such as fecal calprotectin and CRP are the most important effective monitoring tools.^[21,22]^

Fecal calprotectin correlates well with endoscopic disease activity. It is important in a variety of clinical settings, including initial diagnosis, relapse, and response to treatment, whereas CRP correlates less well with clinical severity in ulcerative colitis, except for acute severe colitis.^[15]^ Syrian current humanitarian crisis has been harmed chronic disease patients particularly in terms of medicine availability as many were unavailable, and difficulties in maintaining their presence.^[23–25]^ It is common practice in Syria to evaluate UC patients with clinical scores and CRP levels. If there were signs of treatment failure, we performed a colonoscopy and fecal calprotectin.^[15,26]^ 5-ASA enemas, which are not available in Syria, were used to treat mild to moderate ulcerative colitis on the left side and were preferred over rectal steroids for induction of remission.^[10–12,27,28]^ Immunosuppressive therapy such as steroids, azathioprine, and anti-TNFα play a significant role in treatment.^[10–12]^ Anti-TNFα has the potential to activate opportunistic infections;^[29]^ therefore, it is important to screen for its presence before starting biological treatment such as latent tuberculosis,^[30,31]^ and hepatitis B.^[32]^ However, some opportunistic infections, such as cytomegalovirus, do not require scanning for its presence before starting treatment.^[29,33–35]^ Current guidelines recommend reverse transcription-polymerase chain reaction as a test for COVID-19 before an endoscopic procedure,^[36,37]^ and before initiating long-acting biologic therapy.^[38]^ It has the highest sensitivity and specificity, but it is expensive and can take up to 24 hours to produce results, while rapid antigen tests are becoming more popular due to their low cost and near-instant results.^[39]^ The COVID-19 pandemic is an ongoing global health crisis, causing progressive pneumonia and may be complicated by and multi-organ failure.^[40,41]^ There are conflicting views on the role of immunosuppressants in COVID-19. Some argue that immunosuppressive drugs have a negative effect on the disease's progression.^[42]^ Others argue that immune modulators, both specific and non-specific, can inhibit cytokines and calm the cytokine storm.^[43]^ This disparity could be attributed to the COVID-19, the patients, or the immunosuppressant used. Severe cases of COVID-19 that may lead to death are attributed to, cytokine release syndrome, which is characterized by elevated serum interleukin (IL-6, IL-8, IL-10, and TNFα) levels, cytokine release syndrome-induced macrophage activation syndrome, and hemophagocytic histiocytosis.^[5,9,44–47]^ Comorbidities, old age, and male sex are all risk factors for COVID-19 infection poor outcomes.^[48,49]^ Because of the potential interactions between the immune response associated with COVID-19 and dysfunctional immunity associated with inflammatory bowel disease, patients with both diseases may face unique challenges in their management. In ASUC, we rely on scores such as Mayo, partial Mayo, and Truelove and Witts to assess treatment response.^[15]^ Those scores included parameters such as temperature, diarrhea frequency, and the results of laboratory tests such as CRP and erythrocyte sedimentation rate, which are related to COVID-19 infection. This adds another challenge for assessing and managing patients with ASUC and COVID-19. The treatment of patients with ASUC and COVID-19 is complicated. Current recommendations are to use lower doses of prednisone (20 mg/d) or budesonide and to avoid thiopurines, methotrexate, and tofacitinib, and postpone anti-TNF therapies, ustekinumab, and vedolizumab for 2 weeks while monitoring the development of COVID-19. This will aid in the search for the disappearance of IgM antibodies and the development of IgG antibodies.^[50,51]^ Although chest computed tomography scans aid in the diagnosis of infection, they also help in the assessment and management of patients.^[52–54]^ According to Stallmach et al and Honore et al Infliximab may prevent COVID-19-induced cytokine storm syndrome and reduce mortality in critically ill COVID-19 patients.^[55,56]^ Two case reports have described the use of anti-TNF in the context of inflammatory bowel diseases and COVID-19 infection. Tursi et al reported a case of crohn's disease treated with adalimumab for 5 years. Adalimumab is administered at a dose every 2 weeks, and its scheduled dose is suspended when COVID-19 infection has been proven.^[57]^ While Abdullah et al reported a case of an infliximab ulcerative colitis patient who achieved sustained clinical remission with infliximab and was infected with COVID-19. The patient did not require hospitalization and the clinical symptoms resolved completely within a week of onset, with no change in ulcerative colitis activity. The next dose of infliximab will be approximately 8 weeks later.^[58]^ In our case, several factors influenced the decision to administer the second dose of infliximab, including the indication for infliximab as ASUC refractory to corticosteroids, and the first infliximab dose produced a significant response. It also had no negative effects on COVID-19 infection and infliximab antibodies formation as a result of intermittent treatment, and maintenance therapy is most likely the most effective strategy for optimizing treatment and avoiding immunogenicity.^[59]^

What makes this case unique is that we used 2 doses of infliximab within 2 weeks to treat ASUC with COVID-19 pneumonia; the first dose was 5 days before the onset of COVID-19 symptoms, and the second was 9 days later. Neither dose resulted in serious complications, as the pulmonary symptoms subsided within a week. The patient completed infliximab induction at a dose of 5 mg/kg at weeks 0, 2, and 6, with no complications. Data on anti-TNFα effects in COVID-19 patients are limited, although current treatment guidelines advise against the use of anti-TNFα in patients with acute COVID-19 infection. These recommendations are weak as they are not based on clinical studies. This case demonstrates that infliximab can be safely used. However, further studies are needed to determine the factors involved in the selection of patients to continue biological treatment in association with acute COVID-19.

All authors have read and approved the manuscript on behalf of all contributors I will act and guarantor and will correspond with the journal from this point onward.

## Author contributions

**Conceptualization:** Marouf Mouhammad Alhalabi.

**Project administration:** Marouf Mouhammad Alhalabi.

**Supervision:** Marouf Mouhammad Alhalabi.

**Visualization:** Marouf Mouhammad Alhalabi.

**Writing – original draft:** Marouf Mouhammad Alhalabi.

**Writing – review & editing:** Marouf Mouhammad Alhalabi, Kamal Alaaeddin, Fadwa Ali, Ahmad Abbas.
